# Proteomic profiling of peripheral blood neutrophils identifies two inflammatory phenotypes in stable COPD patients

**DOI:** 10.1186/s12931-017-0586-x

**Published:** 2017-05-22

**Authors:** Adèle Lo Tam Loi, Susan Hoonhorst, Corneli van Aalst, Jeroen Langereis, Vera Kamp, Simone Sluis-Eising, Nick ten Hacken, Jan-Willem Lammers, Leo Koenderman

**Affiliations:** 1 Departments of Respiratory Medicine, University Medical Center Utrecht, Utrecht, The Netherlands; 2 Departments of Respiratory Medicine, University Medical Center Groningen, Groningen, The Netherlands; 3 Department Respiratory Medicine and Laboratory of Translational Immunology, University Medical Center Utrecht, Heidelberglaan 100, 3583CX Utrecht, The Netherlands

**Keywords:** COPD, Proteomics profile, Neutrophil, Systemic inflammation, Inflammatory phenotype

## Abstract

**Background:**

COPD is a heterogeneous chronic inflammatory disease of the airways and it is well accepted that the GOLD classification does not fully represent the complex clinical manifestations of COPD and this classification therefore is not well suited for phenotyping of individual patients with COPD. Besides the chronic inflammation in the lung compartment, there is also a systemic inflammation present in COPD patients. This systemic inflammation is associated with elevated levels of cytokines in the peripheral blood, but the precise composition is unknown. Therefore, differences in phenotype of peripheral blood neutrophils in vivo could be used as a read out for the overall systemic inflammation in COPD.

**Method:**

Our aim was to utilize an unsupervised method to assess the proteomic profile of peripheral neutrophils of stable COPD patients and healthy age matched controls to find potential differences in these profiles as read-out of inflammatory phenotypes. We performed fluorescence two-dimensional difference gel electrophoresis with the lysates of peripheral neutrophils of controls and stable COPD patients.

**Results:**

We identified two groups of COPD patients based on the differentially regulated proteins and hierarchical clustering whereas there was no difference in lung function between these two COPD groups. The neutrophils from one of the COPD groups were less responsive to bacterial peptide N-formyl-methionyl-leucyl-phenylalanine (fMLF).

**Conclusion:**

This illustrates that systemic inflammatory signals do not necessarily correlate with the GOLD classification and that inflammatory phenotyping can significantly add in an improved diagnosis of single COPD patients.

**Trial registration:**

Clinicaltrials.gov: NCT00807469 registered December 11th 2008

## Background

Chronic Obstructive Pulmonary Disease (COPD) is characterized by irreversible airflow limitation [1] and is a leading cause of mortality and morbidity worldwide [2]. Cigarette smoking is the most important risk factor for the development of COPD in the western world. According to the Global Initiative for Chronic Obstructive Pulmonary Disease (GOLD) the diagnosis and severity of COPD is assessed using lung function measurements: Forced Expiratory Volume (FEV1) and Forced Vital Capacity (FVC) [3, 4]. It is well accepted that these spirometry measurements are insufficient, mainly because spirometry data alone poorly correlate with symptoms and health status [5, 6].

Many studies have been focused on the identification of disease phenotypes in COPD and have searched for individual and/or combined biomarkers using data from exhaled breath condensate [7], lung biopsies [8–10], induced sputum [11], plasma [12–14] and bronchoalveolar lavage (BAL) fluid [15]. Unfortunately, bonchoscopy to obtain lung tissue and BAL fluid and to a lesser extent induced sputum are invasive procedures and are therefore difficult to perform regularly in COPD patients especially in the more severe phenotypes. Therefore, there is an unmet clinical need for objective disease markers identifying COPD phenotypes that can be obtained with non invasive methods.

The airflow limitation in COPD is usually progressive and is associated with an abnormal inflammatory response in the lungs. This inflammation in the lung is characterized by an accumulation of neutrophils, macrophages and lymphocytes [16]. Besides the inflammation in the lung there is also a systemic inflammation that is normally illustrated by elevated levels of cytokines (TNFα, IL-8) and CRP in the peripheral blood of COPD patients [17, 18]. Oudijk et al. [19] have shown that peripheral blood neutrophils from COPD patients were characterized by modulated gene expression of IL-1β, IL-1Rα, MIP-1β and CD83 [19], which correlated with a decreased FEV1. We have shown that pro-inflammatory cytokines such as GM-CSF and TNFα can both synergize and antagonize inflammatory signaling pathways in neutrophils [20]. Therefore, measurements of individual cytokines using multiplex assays without determining their natural modulators in clinical samples could lead to wrong conclusions. And, therefore, modulation of protein expression in peripheral blood neutrophils as read-out of the combined action of pro- and anti-inflammatory cytokines on leukocytes could be a better approach to measure systemic inflammation in COPD patients.

We collected peripheral blood neutrophils from COPD patients to perform proteomics analysis. We hypothesize that the net result of multiple cytokines on the change in phenotype of peripheral blood neutrophils in vivo can be used as a read out for the overall inflammatory status of the COPD patients. We used the unbiased fluorescence two-dimensional (2D) difference gel electrophoresis (DIGE) method to analyze protein expression of peripheral blood neutrophils from COPD patients and healthy aged matched controls.

## Methods

### Patients and healthy controls

We included 41 stable COPD patients (age 40–75 years) with a smoking history of more than 10 pack years and a ratio of FEV1 to forced vital capacity (FVC) below 70% after bronchodilator use, as described by the global initiative for chronic obstructive lung disease (GOLD) [21]. COPD patients with a history of other inflammatory diseases, acute pulmonary disease or other infections, treatment with antibiotics or corticosteroids 8 weeks to inclusion, recent diagnosis of cancer and a history of asthma were excluded. Control subjects were healthy age-matched subjects with normal lung function and no medical history of pulmonary disease. This study was approved by the medical ethical committee and all study subjects provided written informed consent. This study is based on data collected as part of a bi-center cross-sectional study performed by University Medical Centers of Utrecht and Groningen, with trial register numbers NCT00850863 and NCT00807469 (www.clinicaltrials.gov).

### Reagents and antibodies

Ficoll-Paque was obtained from GE Healthcare (Uppsala, Sweden). Human Serum Albumin (HSA) was from Sanquin (Amsterdam, the Netherlands). HEPES-buffered RPMI 1640 was from Invitrogen (Carlsbad, CA, USA). Dihydrorhodamine 123 (DHR) was purchased from Sigma Aldrich (St. Louis, MO, USA) and dissolved in DMSO at a concentration of 3,33 mg/ml and stored at −20 °C. Platelet Activating Factor (PAF) and fMLF were purchased from Sigma Chemical Co (St. Louis, MO, USA). The VIM12 (CD11b, Mac-1, IgG1) was obtained from Caltag Invitrogen (Carlsbad, CA, USA). For flow cytometry staining we used the antibodies CD18 (clone L130); CD15 (clone MMA); CD32 (clone FLI8.26), CD35 (clone E11), CD44 (clone 515), CD63 (clone H5C6) and CD16 (clone 3G8) obtained from BD Pharmingen (San Diego, CA, USA). CD11b (clone 2LPM19c) was from DAKO (Copenhagen, Denmark), CD66b (clone 80H3) was from Cytognos (Salamanca, Spain), CXCR1 (clone 42705), CXCR2 (clone 48311) and CD45 (clone 2D1) were from R&D systems (Europe, UK) and CD64 (clone 10.1) was from AbD Serotec (Oxford, UK). CD29 (clone N29) was purchased from Millipore. All other chemicals were reagent grade.

### Granulocyte isolation

Granulocytes were isolated from whole blood anticoagulated with sodium-heparin from COPD patients or age-matched healthy control subjects. Blood was diluted 2.5:1 with PBS containing trisodium citrate (0.4% *w/v*, pH 7.4) and human pasteurized plasma-protein solution (4 g/L). The granulocytes were separated from the mononuclear cells by centrifugation using Ficoll-Paque. Erythrocytes were lysed in isotonic ice-cold NH_4_Cl solution (8.3 g/L NH_4_Cl, 1 g/L KHCO_3_ and 37 mg/L EDTA) followed by centrifugation at 4 °C. After isolation, granulocytes were washed in PBS containing trisodium citrate (0.4% *w/v*, pH 7.4) and human pasteurized plasma-protein solution (4 g/L) and resuspended in HEPES buffered RPMI 1640 supplemented with 0.5% (*w/v*) HSA. Purity of neutrophils was >95% with eosinophils as major contaminant.

### Protein extract preparation

Neutrophils (5 × 10^6^/mL) in HEPES buffered RPMI 1640 supplemented with 0.5% (w/v) HSA were incubated for 15 min at 37 °C. Subsequently, neutrophils of COPD patients and healthy age-matched controls were immediately prepared for protein extracts. The neutrophils (1 × 10^7^/sample) were washed twice with sucrose buffer (0.34 M sucrose, 1 mM EDTA, 10 mM Tris) and lysed in lysis buffer (10 mM Tris pH 7.4, 10% glycerol, 1% NP40, 50 mM NaF, 20 mM tetra-Na pyrophosphate, 1 mM DTT, 2 mM vanadate, 1 mM PMSF, 2 mM DFP and 1 × Complete EDTA-free protease inhibitor cocktail tablet (Roche)). Proteins were precipitated with 80% acetone and dissolved in labeling buffer (8 M Urea, 2 M Thiourea, 4% CHAPS, 10 mM Tris pH 8.5).

### CyDye labeling

The DIGE technology is an unbiased approach to identify differences in protein expression and the use of an internal standard enables identification of protein differences as small as 10% [22]. Protein extracts were labeled using the fluorescent cyanine dyes developed for 2D-DIGE technology (GE Healthcare) following manufacturer’s protocol. Protein extracts (30 μg) were labeled with 300 pmol of fluorescent dye (Cy2 or Cy3 or Cy5). Protein samples from COPD patients and healthy age-matched control were randomly labeled with Cy3 or Cy5. To exclude the effects of preferential labeling each dye was used a similar number of times in each group. An internal standard, created by pooling 15 μg of each protein sample, was labeled with Cy2. Labeling was stopped by adding lysine and equal volume of 2 × IEF buffer (8 M Urea, 2 M Thiourea, 4% CHAPS, 300 mM DTT, 1.0% IPG buffer 3-10NL, 0.004% Bromophenolblue) to each sample.

### 2D-gel electrophoresis and analysis

Two protein samples (Cy3 and Cy5) were mixed with the Cy2-labeled internal control. Protein samples were passively rehydrated into 24 cm pH 3-10 NL strips (GE Healthcare, Uppsala, Sweden) for 10 h followed by isoelectric focusing using a manifold-equipped IPGphor IEF unit (GE Healthcare) according to the manufacturer’s protocol. The cysteine sulfhydryls were reduced with 1.0% DTT and carbamidomethylated with 2.5% Iodoacetamide in equilibration buffer (30% glycerol, 2% SDS, 6 M urea, 75 mM Tris, pH 8.8). Second dimensional SDS-PAGE was performed on hand-cast 12% SDS-PAGE gels using low fluorescence glass plates. Electrophoresis was carried out at 0.2 watts/gel for 2 h followed by 1 watts/gel until completion using an Ettan DALT-12 unit (GE Healthcare). Gels were scanned using a Typhoon 9410 imager at 100 μm resolution (GE Healthcare). Scan settings were optimized for a maximal signal of 85.000 counts. The gel images were cropped using ImageQuantTL 2003 (GE Healthcare), spot detection was performed with DeCyder 7.0 DIA (Difference In-gel Analysis) software (GE Healthcare) and gel images were matched using DeCyder 7.0 BVA (Biological Variation Analysis) software (GE Healthcare). Proteins present in >70% of the spot maps were included in the analysis and proteins with an average ratio of at least 1.5 and *p* < 0.05 were considered significant.

### Flow cytometry analysis

**Fig. 1:**
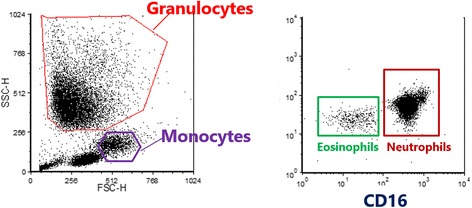
Flowcytometry gating strategy. Granulocytes and monocytes were identified according to forward and sideward scatter signal. Neutrophils and eosinophils were identified according their CD16 expression

Blood was collected from COPD patients in sterile collection tubes containing sodium heparin as an anti-coagulant. The erythrocytes from the whole blood were lysed with ice-cold NH_4_Cl. The leukocytes were washed once with ice cold NH_4_Cl and centrifuged at 4 °C. Hereafter, the cells were washed and resuspended in PBS buffer for FACS analysis in (PBS containing 0.32% (*w/v*) sodium citrate and human pasteurized plasma solution (4 g/L)). Unstmulated leukocytes (1.25 × 10^5^ cells) were stained with CD11b, CD18, CD15, CD66b, CXCR1, CD64, CD29 and CD32 in combination with CD16 for 30 min at 4 °C, washed once in PBS buffer and resuspended in PBS buffer. Cells were analyzed on a FACs Calibur flow-cytometer (Becton Dickinson, Mountain View, CA, USA). Neutrophils were identified according to their specific side scatter and forward scatter signals and CD16 expression (for the gating strategy see Fig. 1). The experiments were controlled by staining cells with isotype control antibodies (see e.g. Fig. 5).

For staining with antibodies against the active form of FcγRII we used the directly FITC-labelled monoclonal phage antibody A17, as described previously [23]. In short, whole blood was stimulated in the presence or absence 10 ^-6^ M of fMLF for 5 min at 37 °C. After stimulation the A17 antibody and CD11b PE antibody were added to 50 μl whole blood and incubated for 60 min at 4 °C. After staining erythrocytes were lysed and expression of active FcγRII (A17) and CD11b were measured on a FACS Calibur (Becton Dickinson, Mountain View, CA, USA).

### Respiratory burst assay

After lysis of the erythrocytes, the leukocytes were washed and resuspended in HEPES buffer (20 mM HEPES, 132 mM NaCl, 6 mM KCl, 1.2 mM KH_2_PO_4_, 1 mM MgSO_4_), supplemented with 5 mM glucose, 1 mM CaCl_2_, and 0.5% (*w/v*) human serum albumin (HSA). The leukocytes (2.0 × 10^6^ cells/ml) were primed with 10 ^-6^ M PAF at 37 °C. After 2 min DHR was added to a final concentration of 1 μMol/L and incubated for 5 min. In some experiments the cells were stimulated with fMLF (10 ^-6^ M) or VIM12 (10 μg/ml) at 37 °C in the dark. After stimulation the cells were washed with 3 ml of cold PBS supplemented with 1% (*w/v*) HSA and samples were kept at 4 °C in the dark. The production of reactive oxygen species (ROS) was analyzed with a FACs Calibur flow cytometer (Becton Dickinson, Mountain View, CA, USA). Neutrophils were identified by their specific side scatter and forward scatter signals.

### Multiplex analysis

Plasma from COPD patients and healthy age-matched controls were obtained from blood anti-coagulated with EDTA after immediate centrifugation (2000 g at 4 °C for 10 min) and were stored at −80 °C until analysis. Concentrations of cytokines were determined by applying the Luminex platform as has been described before [24].

### Statistical analysis

Statistical analysis of 2D-DIGE spot intensity was performed using DeCyder 7.0 BVA or EDA software (GE Healthcare, Uppsala, Sweden). Other statistical analyses were performed if appropriate using either an independent sample *t* tests or an ANOVA with statistical software package IBM SPSS 20.

## Results

### Patient characteristics

In total, 41 at inclusion stable COPD patients and 7 healthy age-matched controls were included in this study. Fourteen COPD patients were currents smokers, whereas 27 of the COPD patients were ex-smokers. Four of the healthy controls were never smokers and 3 were ex-smokers. Details of the COPD patients and the controls are described in Table 1. It is important to emphasize that this study was set up to identify phenotypes within our group of stable COPD patients and not to study differences between COPD patients and healthy individuals. No statistical differences were found for age, BMI or hsCRP; whereas the FEV1 and the FEV1/FVC were statistically different between the COPD patients and the controls. Although the leukocyte counts of the COPD patients were not significantly different from the controls, the COPD patients have a higher leukocyte count than the controls (*p* = 0.05). This could implicate that there is an ongoing but low systemic inflammation in the COPD patients.

**Table 1:** Patient characteristics

Characteristics	Controls (*N* = 7)	COPD (*N* = 41)	Statistics
Age, yrs	60 ± 6	60 ± 8	0.929
Gender			
Male	5	30	
Female	2	11	
FEV1, L	3.4 ± 1.0	1.6 ± 1.0	< 0.001
FEV1, % predicted	109 ± 21	50 ± 26	< 0.001
FEV1/FVC ratio	77 ± 4	41 ± 13	< 0.001
GOLD			
I		8	
II		8	
III		12	
IV		13	
MRC dyspnoe scale			
0	7	1	
1		10	
2		12	
≥ 3		18	
Smoking status			
Current smoker		14	
Ex-smoker	3	27	
Never smoker	4		
BMI, kg/m2	24 ± 1.4	24 ± 3.3	0.790
hsCRP	3.8 ± 5.4	4.6 ± 6.9	0.765
Leukocyte count	6.2 ± 1.9	7.7 ± 1.8	0.052

Continues data are shown as mean ± SD

*FEV1* Forced Expiratory Volume 1, *FVC* Forced Vital Capacity, *MRC* Medical Research Council, *GOLD* Global Initiative for Chronic Obstructive Pulmonary Disease, *BMI* Body mass index

### 2D-DIGE analysis of peripheral neutrophils from controls and COPD patients

**Fig. 2:**
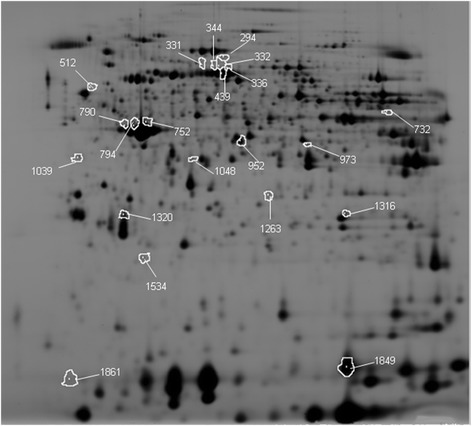
2D-DIGE gel with the 21 differentially expressed proteins. Protein samples were focused on 24 cm pH3-10NL IEF strips and separated by 12% SDS-PAGE. The indicated spots represent the differentially regulated proteins identified by Decyder 7.0 analysis software

**Fig. 3:**
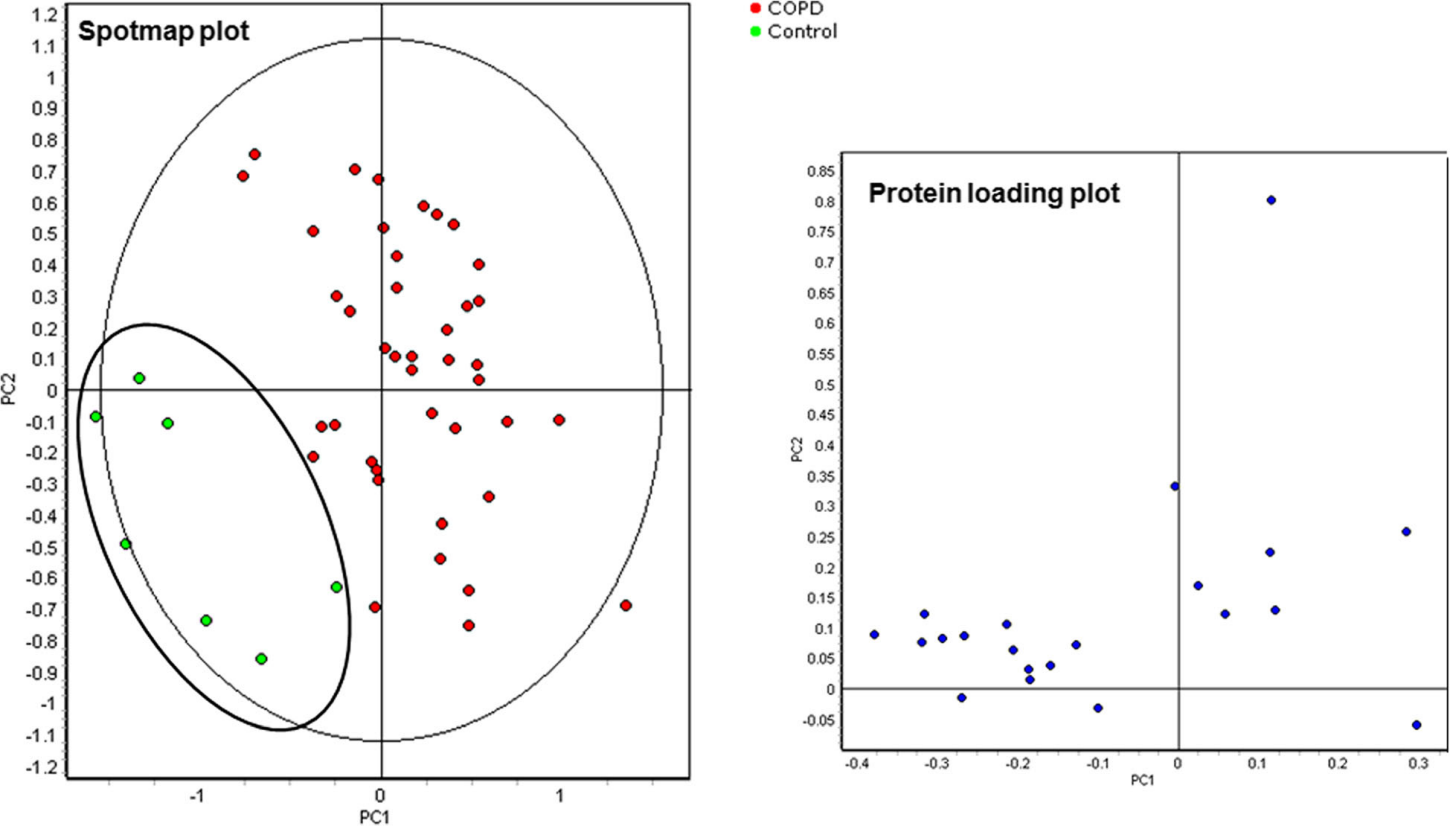
COPD spot maps were discriminative in a principal component analysis. Data of the 21 significant differentially regulated proteins (*blue spots* in the loading plot) in the Biological Variation Analysis (BVA) of peripheral neutrophils from COPD patients were imported in the extended data analysis (EDA) software. Principal component analysis (PCA) was performed on 41 COPD (*red dots*) and 7 age-matched control (*green dots*) spot maps

We performed 2D-DIGE with peripheral neutrophils isolated from peripheral blood of healthy age matched controls and COPD patients. All protein samples were labelled with CyDye and separated by 2D-DIGE in different experiments. Upon analysis 1200–2400 protein spots were identified by the DeCyder 7.0 software. Here a volume filter exclusion was used of 30 000 in the Difference In-gel Analysis (DIA) software. The individual spot maps were matched in the Biological Variation Analysis (BVA) Software. We matched 2058 protein spots, of which 875 proteins were present in >70% of the spot maps. Statistical analysis between healthy age-matched controls and COPD patients showed 21 proteins spots that were at least 1.5 fold differentially regulated with a *p* < 0.05 (Fig. 2). Based on these 21 differentially regulated proteins the peripheral neutrophil spot maps from COPD patients could be separated from healthy age-matched controls by principal component analysis (PCA) (Fig. 3).

### Hierarchical clustering of COPD and healthy control samples based on differential protein expression

**Fig. 4:**
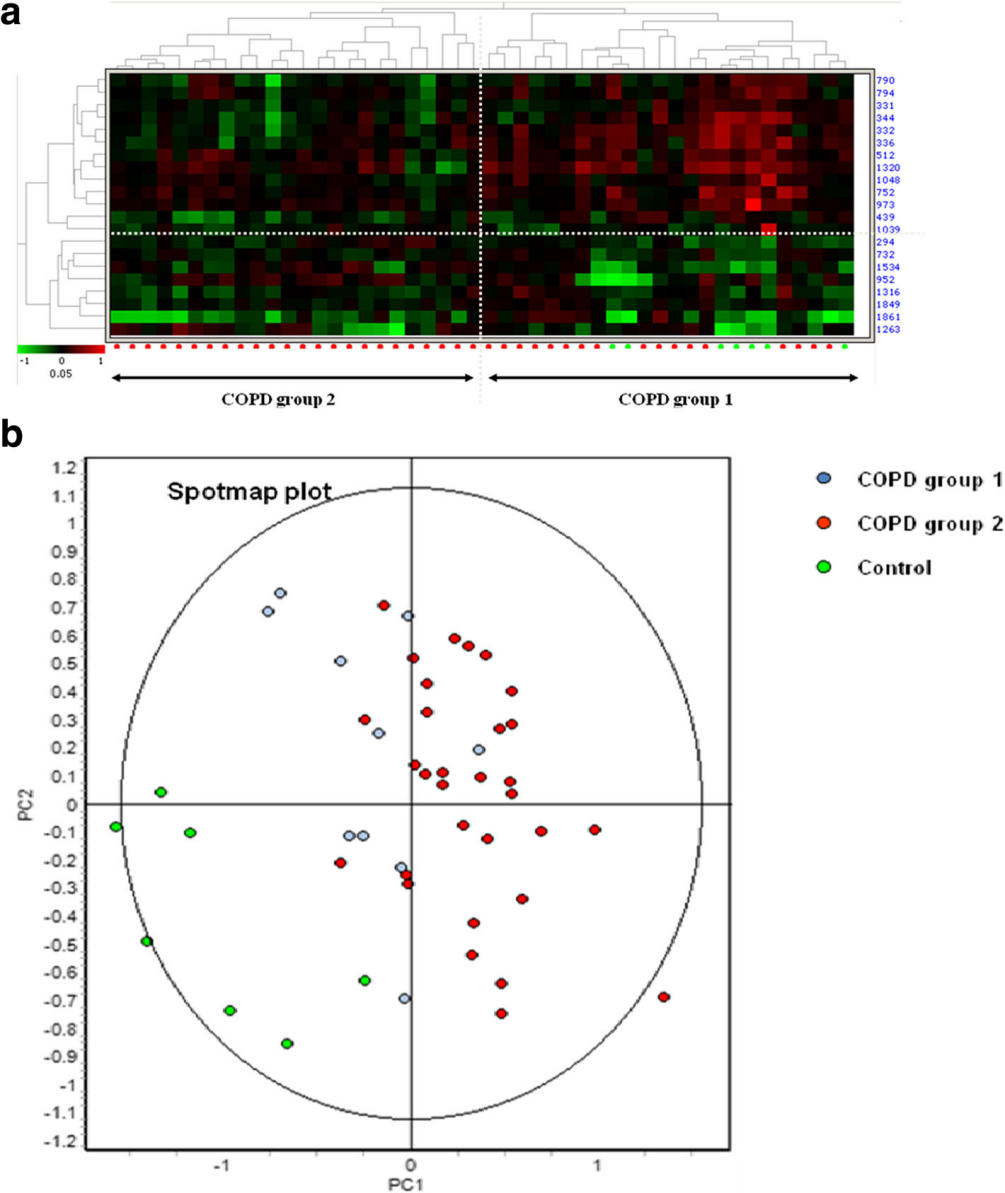
**a**-**b** Hierarchical clustering control and COPD spot maps and principal component analysis. Twenty one differentially regulated proteins were identified with the Decyder 7.0 analysis software and used for hierarchical clustering in the extended data analysis (EDA) of the Decyder 7.0. Clustering of the differentially regulated protein expressed (*red* is up-regulated, green is down-regulated) revealed two groups of spot maps. Group 1 were mixed COPD and age-matched healthy control spot maps and Group 2 solely comprised COPD spot maps. The COPD spot maps in group 1 were designated as COPD group 1 and the COPD spot maps in group 2 were designated as COPD group 2 (**a**). The COPD group 1 spot maps in the Principal component analysis based on the 21 significant differentially regulated proteins were depicted in *blue*. Whereas the COPD group 2 spot maps were depicted in *red* and the age-matched controls were depicted in green (**b**)

We observed differential protein expression in peripheral blood neutrophils between COPD patients and healthy age-matched controls using 2D-DIGE. We combined all differentially regulated proteins using the EDA in the DeCyder 7.0 software package and performed a hierarchical clustering analysis. Based on the 21 differentially expressed proteins we observed two groups in this way (Fig. 4a). COPD group 1 consists of a mix of healthy age-matched control and COPD spot maps, whereas COPD group 2 comprised solely COPD spot maps. The COPD patients which were mixed with the healthy age-matched controls (COPD group 1) in the hierarchical clustering were indicated (blue dots) in the PCA analysis of the 21 differentially expressed proteins (Fig. 4b).

### No differences in clinical parameters between COPD group 1 and COPD group 2

The hierarchical clustering showed a group that resembled neutrophils of healthy age-matched controls (COPD group 1) and a distinct group of COPD patients (COPD group 2) based on their differential protein expression. When we compared the lung function data (FEV1, FEV1/FVC) between COPD patients in group 1 with the patients in group 2 there were no significant differences (Table 2). Therefore, the difference in peripheral neutrophil proteome identified by hierarchical clustering of spot maps did not correspond to the current classification of COPD based on FEV1 and FEV1/FVC. Also the hsCRP values and the total leukocyte counts were not significantly different between the two COPD groups (Table 2). Other clinical parameters are described in Table 2; these were also not significantly different between the two groups of COPD patients.

**Table 2:** Clinical data of COPD group 1 and COPD group 2

Clinical parameters	COPD group 1 (*N* = 17)	COPD group 2 (*N* = 24)	Statistics
Age, yrs	59 ± 6	61 ± 9	0.367
FEV1, L	1.89 ± 1.27	1. 40 ± 0.69	0.167
FEV1, % predicted	56 ± 30	46 ± 22	0.259
FEV1/FVC ratio	44 ± 15	39 ± 12	0.214
Pack years	46 ± 18	38 ± 14	0.160
DLCO	56 ± 20	60 ± 21	0.531
P15, HU	−946 ± 23	−953 ± 22	0.392
6MWD, m	472 ± 106	476 ± 131	0.904
Pack years	46 ± 18	38 ± 14	0.160
MRC dyspnoe scale	2.2 ± 1.1	2.6 ± 1.4	0.414
CCQ total score	2.1 ± 1.2	1.9 ± 1.1	0.696
SGRQ total score	44 ± 19	44 ± 17	0.899
BODE index	3.1 ± 2.7	3.4 ± 2.5	0.718
hsCRP	4.3 ± 5.4	4.9 ± 7.8	0.779
Leukocyte count	7.7 ± 1.6	7.7 ± 2.0	0.931

*FEV1* Forced Expiratory Volume 1, *FVC* Forced Vital Capacity, *DLCO* Diffusion capacity of the lung for carbon monoxide, *P15* 15th percentile, *HU* hounsfield unit, *6MWD* 6 min walking distance, *CCQ* Clinical COPD Questionnaire, *SGRQ* St. George’s Respiratory Questionnaire

### Single activation markers on peripheral neutrophils are not significantly different between COPD group 1 and COPD group 2

**Fig. 5:**
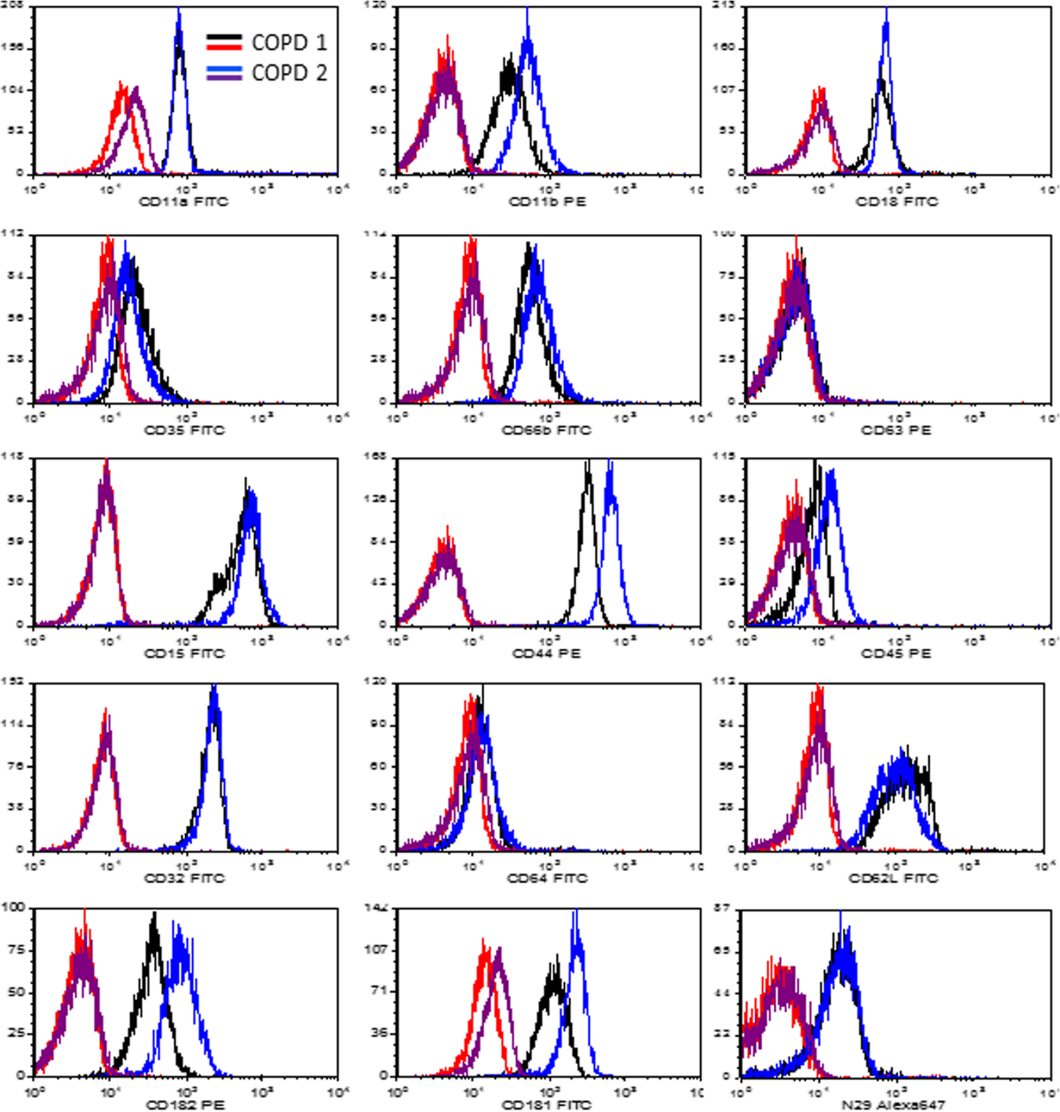
Histogram overlays of the expression of all used markers on neutrophils from COPD group 1 and COPD group 2. Representative example of the subtle changes in expression of different markers on neutrophils isolated from patient blood of the different COPD groups. Isotype controls are shown in *red* and *purple* for cells of COPD 1 and COPD 2, respectively. For statistics see Table 3

Although the neutrophils proteome was different between the two COPD groups; the expression of several activation markers were not significantly different (Table 3 and Fig. 5). The expression was measured on native non-activated neutrophils. The activation markers Mac-1, CD66b and CXCR1 were similar between the two groups. Although the active FcγRII appeared to be higher in COPD group 2, it did not reach statistical significance.

**Table 3:** The activation markers on (unstimulated) peripheral neutrophils between COPD group 1 and COPD group 2

Activation markers	COPD group 1 (*N* = 17)	COPD group 2 (*N* = 22)	Statistics
MFI	Mean ± SD	Mean ± SD	
CD11b	85 ± 36	78 ± 64	0.699
CD18	96 ± 67	80 ± 31	0.371
CD64	38 ± 65	27 ± 31	0.493
CD66b	75 ± 36	78 ± 34	0.762
Active FcγRII	65 ± 68	120 ± 239	0.367
CD32	214 ± 52	233 ± 34	0.136
CXCR1	190 ± 58	188 ± 49	0.936
CD62L	255 ± 234	194 ± 75	0.317
CD45	10 ± 4.86	11 ± 4.12	0.541
CXCR2	64 ± 17	67 ± 30	0.920
CD35	31 ± 15	32 ± 21	0.310
CD15	958 ± 363	1171 ± 519	0.920
CD63	4.02 ± 0.78	4.46 ± 1.42	0.920
CD44	541 ± 114	579 ± 163	0.152
N29	17 ± 4	19 ± 11	0.920

*MFI* median fluorescence intensity, *SD* standard deviation

### Differences in cytokine levels between COPD group 1 and COPD group 2

**Fig. 6:**
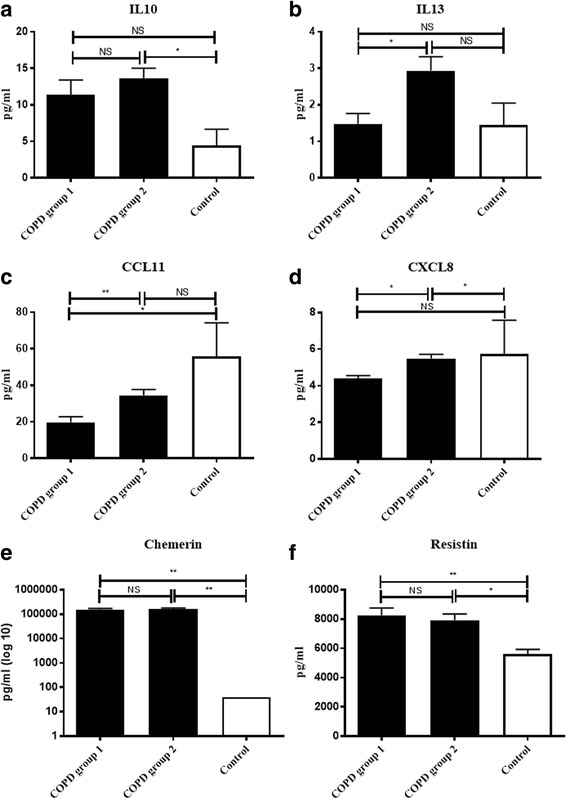
Cytokine differences between the two COPD groups and the age-matched controls. Plots with the mean plasma levels of IL10, IL13, CCL11, CXCL8, Chemerin and Resistin (**a**-**f**) in age-matched controls (*n* = 6), COPD patient group 1 (*n* = 17) and COPD patient group 2 (*n* = 24). Cytokine levels are expressed as Mean ± SEM. An ANOVA was used to perform statistics (* *p* < 0.05; ** *p* < 0.01 NS = not significant)

We measured the levels of a number of cytokines but only a few were significantly different between the two COPD groups or between COPD and healthy age-matched controls (Fig. 6). The cytokine IL13 and the chemokines CCL11 and CXCL8 were significantly different between COPD group 1 and 2 (Fig. 6b–d), whereas IL10 and CXCL8 (Figs. 4d, 6a) were significantly different between COPD group 2 and the healthy age-matched controls. The cytokines CCL11, chemerin and resistin (Fig. 6c, e–f) were different between COPD group 1 and the healthy age-matched controls.

### Reduced ROS production in COPD group 2

**Fig. 7:**
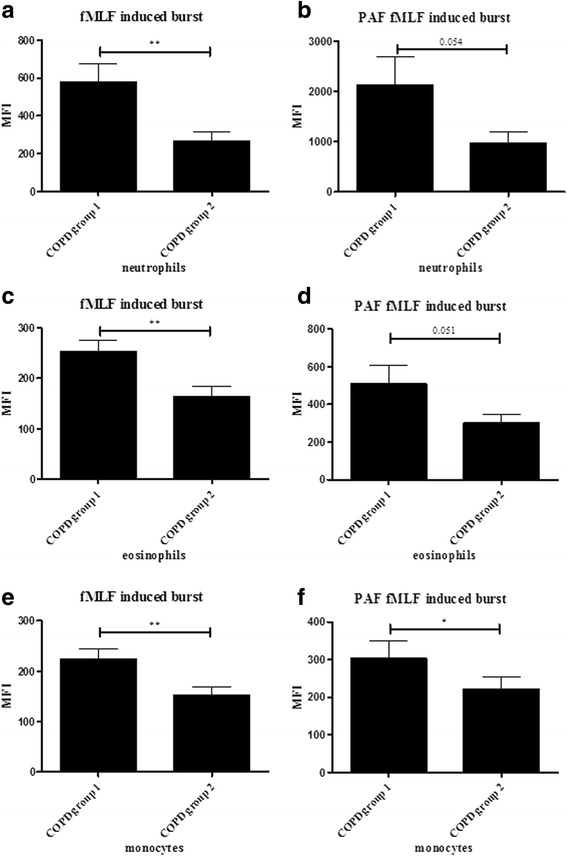
The respiratory burst (ROS production) assay. Leukocytes (2.0 10^6 cells/ml) of age-matched controls (*n* = 12), COPD patient groups 1 (*n* = 17) and COPD patient group 2 (*n* = 19) were pre-incubated in the presence or absence of PAF (10^−6^ M); loaded with DHR 123 for 5 min and stimulated with fMLF (**a**-**f**) for 15 min at 37 °C. Production of ROS was measured by oxidation of intracellular DHR 123 and measured using flow cytometry (Mean ± SEM). An independent *t*-test (** = *p* < 0.01, * = *p* < 0.05) was used to perform statistical analysis

The peripheral neutrophils from COPD group 2 had lower respiratory burst (ROS production) compared to the neutrophils from COPD group 1 (Fig. 7a–b). This was also the case for eosinophils (Fig. 7c–d) and monocytes (Fig. 7e–f). The respiratory burst of peripheral neutrophils after fMLF stimulation in controls was higher compared to COPD group 2 and lower compared to COPD group 1 (Fig. 7a–b).

## Discussion

It has been well accepted that there is only a very poor relation between the GOLD stages and clinical manifestations such as symptoms and the health status of COPD patients [5, 6]. COPD is a heterogeneous disease that is characterized by both pulmonary and extra-pulmonary manifestations and the GOLD classification does not include the extra-pulmonary components of this disease. We tested the hypothesis that multiple COPD phenotypes exist and that these are characterized by differences in systemic inflammation. The biological effect and the role in systemic inflammation of single cytokines is difficult to objectify, because of the fast changes in their dynamics/kinetics in the peripheral blood. Also functional cross-talk between different cytokines has been observed, which makes the effect of a pool of cytokines even more difficult to predict. We have shown that pro-inflammatory TNFα can induce enhanced CD83 expression on human neutrophils and that another pro-inflammatory cytokine, GM-CSF, can antagonize this effect [20]. Therefore, we used differences in peripheral neutrophils as a read out for systemic inflammation as these cells integrate pro- and anti-inflammatory signals into a specific activation phenotype. We applied the unbiased proteomics approach to define these activation phenotypes. We compared protein expression of peripheral neutrophils from 41 COPD patients and 7 healthy age-matched controls using our 2D-DIGE approach. With the use of the DeCyder 7.0 software we were able to identify 21 differentially regulated proteins (>1.5 fold and *p* < 0.05) (Fig. 2). Based on the differential expression of these proteins we performed a principle component analysis (PCA) and a hierarchical clustering. There is a clear distinction between the neutrophil proteome of the healthy age-matched controls and the COPD patients, albeit not the focus of this study (Fig. 3). Our data replicate our earlier results showing similar differences [25] in spot maps of neutrophils from a small group of COPD patients and age-matched controls.

A limitation in our study is that about 60% of the healthy age-matched controls were never smokers; whereas COPD is a smoking related disease. In our earlier study [25] spot maps from COPD patients were compared with control spot maps of either ex-smokers or non-smokers in a PCA and still showed clustering of COPD spot maps apart from the control spot maps. Apparently, COPD specific signals could be identified irrespective of the smoking habits of the control groups.

Using the differentially expressed proteins in a hierarchial clustering, we could identify two different clusters. One cluster only existed of COPD patients while the second cluster coexisted of both COPD patients and age-matched controls (Fig. 4a). The existence of the two COPD groups identified by the 2D-DIGE approach suggest that peripheral neutrophils were differentially affected by systemic inflammatory processes in vivo, probably by different combinations of pro-and anti-inflammatory mediators.

We sought to find differences in clinical and immunological parameters between the two COPD groups. We were unable to find statistical differences in lung function (FEV1, FEV1/FVC) (Table 2). Therefore, it is unlikely that the lung function was related to the proteome of the peripheral neutrophils. This fits with other studies including ECLIPSE [26, 27] that failed to demonstrate a correlation between systemic inflammation and lung function in at inclusion stable COPD patients. Interestingly hsCRP, which is known as a marker for systemic inflammation, was not statistically different between the two COPD groups and/or GOLD stages. This is also in line with previous studies [28, 29]. These data add to a growing understanding that if systemic inflammation is an important constituent of the pathogenesis of stable COPD, this process cannot be adequately determined by either GOLD classification nor changes in hsCRP levels. In contrast to stable COPD, the situation with exacerbations is different as high CRP levels have been found in COPD patients under these conditions [30–32].

Our data regarding the absence of differences in expression of CD11b and CXCR1 on neutrophils (Table 3) contrast findings published by others [33, 34]. Although the COPD patients in the studies of Yamagata et al. [34] and Noguera et al. [33] were stable, the expression of CD11b and CXCR1 on neutrophils was measured on isolated neutrophils. Kuijpers et al. [35] have shown that isolation induce activation of neutrophils, which is likely more pronounced on primed cells such as found in our COPD patients (see below). Others have also been unsuccessful in finding enhanced levels of hsCRP in stable COPD patients [28, 29]. Therefore, it seems that a low grade systemic inflammation present in stable COPD patients did not lead to an increased expression of CD11b, CXCR1 or hsCRP.

The systemic inflammation in our COPD cohort was associated with minor differences in the presence of systemic mediators (Fig. 6). We first focused on changes in serum TNFα as differences in the presence of this cytokine has been found previously [17, 36, 37]. We failed to detect differences in levels of TNFα between the controls and the COPD patients which were also not found in ECLIPSE. In this study TNFα appeared to be primarily a marker of smoking and not of COPD [27]. In addition, we found no difference in TNFα between the two COPD groups (data not shown). These data question an important role of systemic TNFα in the pathogenesis of stable COPD. This conclusion is supported by our previous study that [25] showed that the proteomes of peripheral neutrophils of COPD patients were not similar to peripheral neutrophils that were stimulated in vitro by TNFα. The only differences in cytokine levels between the two COPD groups were found for IL13 and chemokines CCL11/eotaxin, CXCL8/IL-8 (Fig. 6b–d). This might indicate that both the systemic eosinophils (IL-13/Eotaxin) as well as the systemic neutrophil (CXCL8/IL-8) compartment are affected in stable COPD without changes in the aforementioned characteristics of the cells.

The above mentioned data prompted us to analyse more subtle changes in granulocyte physiology and analysed the functionality of the cells in the context of activation of the respiratory burst. Neutrophils carefully isolated from normal control individuals are characterized by a low fMLF-induced respiratory burst as this response needs priming by agonists such as PAF that by themselves are poor activators of this response. The relative fMLF response in isolated cells compared to PAF primed cells is a measure of neutrophil priming [38]. The respiratory burst (ROS production) of the peripheral neutrophils of the two COPD groups were significantly different (Fig. 7a–b). The fMLF-induced ROS production of peripheral neutrophils from COPD group 1 patients tended to be higher compared to controls; whereas the ROS production of peripheral neutrophils from COPD group 2 patients was lower. This difference in functionality after fMLF stimulation between COPD group 1 and 2 was not only present in peripheral neutrophils but also in eosinophils and monocytes (Fig. 7c–f). These data fit the hypothesis that COPD group 1 is characterized by the presence of systemic neutrophils with a primed phenotype (higher ROS production compared to healthy controls) and COPD group 2 is characterized by cells with a hypo-responsive phenotype. The underlying mechanisms might be that COPD group 2 has systemic neutrophils with a hypo-responsive phenotype, because hyper-responsive/primed cells have migrated to the tissues leaving behind more refractory cells in the circulation. However, this difference seen in the functionality of the cells did not lead to differences in clinical manifestations, which were measured in this study. More clinical data, better and more subtle differences in innate immune cells, larger cohorts of COPD patients and the identification of the 21 differentially expressed proteins will probably improve the understanding of different COPD phenotypes and the differences found in the functionality of peripheral neutrophils between the two groups.

## Conclusion

We identified a distinct group of COPD patients, who were different from healthy age-matched controls and other COPD patients based on the proteome of peripheral neutrophils. Lung function was not different between the two COPD patient groups. However, the ROS production of peripheral neutrophils was different in COPD group 1 compared to COPD group 2. These data are consistent with the hypothesis that COPD patients are characterized by subtle differences in systemic inflammation that cannot be identified by classical markers. These differences are, however, apparent when the functionality of the cells is studied. Therefore, future studies on targeting systemic inflammation in *stable* COPD should focus on these subtle difference and this difference should not rely only on the classical markers such as individual cytokines, acute phase proteins or single activation markers.

## Abbreviations

BAL: Bronchoalveolar lavage
BVA: Biological variation analysis
CD: Cluster of differentiation
COPD: Chronic obstructive pulmonary disease
Cy: Cyanine
DHR: Dihydrorhodamine
DIA: Difference in-gel analysis
DIGE: Difference gel electrophoresis
EDA: Enhanced data analysis
FEV1: Forced expiratory volume in 1 second
fMLF: Formyl-methionyl-leucyl-phenylalanine
FVC: Forced vital capacity
GM-CSF: Granulocyte-macrophage colony-stimulating factor
GOLD: Global initiative for chronic obstructive pulmonary disease
hsCRP: High sensitiveC-reactive protein
IL: Interleukin
PAF: Platelet-activating factor
PBS: Phosphate-buffered saline
ROS: Reactive oxygen species
TNF: Tumour necrosis factor
